# Neuropathology of neurocutaneous melanosis: histological foci of melanotic neurones and glia may be undetectable on MRI

**DOI:** 10.1007/s00401-012-0945-0

**Published:** 2012-02-01

**Authors:** Veronica A. Kinsler, Simon M. L. Paine, Glenn W. Anderson, D. Saraji Wijesekara, Neil J. Sebire, Wui K. Chong, William Harkness, Sarah E. Aylett, Thomas S. Jacques

**Affiliations:** 1 Department of Paediatric Dermatology, Great Ormond Street Hospital for Children NHS Trust, London, UK; 2 Department of Histopathology, Great Ormond Street Hospital for Children NHS Trust, London, UK; 3 Department of Neuroradiology, Great Ormond Street Hospital for Children NHS Trust, London, UK; 4 Department of Neurosurgery, Great Ormond Street Hospital for Children NHS Trust, London, UK; 5 Department of Neurology, Great Ormond Street Hospital for Children NHS Trust, London, UK; 6 Clinical and Molecular Genetics Unit, UCL Institute of Child Health, 30 Guilford Street, London, WC1N 1EH UK; 7 Neural Development Unit, UCL Institute of Child Health, 30 Guilford Street, London, WC1N 1EH UK

Neurocutaneous melanosis (NCM) is the association of congenital melanocytic naevi (CMN) and melanotic lesions in the central nervous system. The original post-mortem description in 1861 was of progressive proliferative leptomeningeal melanocytosis [17], and until the advent of magnetic resonance imaging (MRI), NCM was thought of as universally fatal. With the recognition of a characteristic MR signal for melanin [1], it is now recognised that a significant proportion of individuals with CMN have CNS involvement in some form [9, 14]. Most commonly, the melanosis appears radiologically as foci of melanin within the brain parenchyma, favouring the amygdala, thalamus, cerebellum and pons [10, 14]. Leptomeningeal melanosis is much less frequent.

Although intra-parenchymal melanosis on MRI is associated with an increased risk of neurological complications [9, 12, 14, 18], a significant proportion of children with CMN have abnormal clinical neurology with normal scans [4, 14, 18]. We report novel neuropathological findings in a case of NCM that support the hypothesis that there may be a wider abnormality of the brain in individuals with CMN, which are undetectable on MRI.

**Fig. 1:**
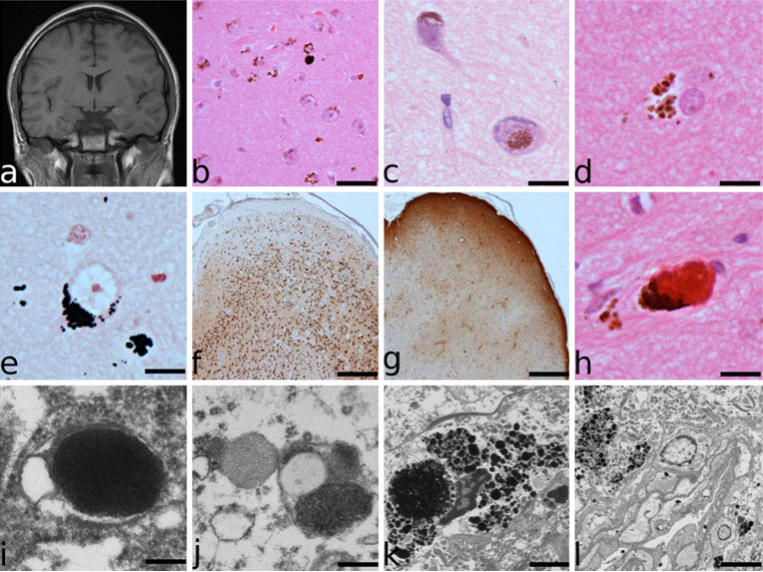
Pre-operative MR images **a** showing T1 shortening, indicating melanin, in the left amygdala. The adjacent neocortex is radiologically normal. Brown melanin pigment was present within the cytoplasm of neurones and astrocytes as well as free in the neuropil but nests of melanocytes were not seen (**b**, H&E, *bar* 50 μm). In the amygdala, pigment was also present in neurones (**c**, H&E, *bar* 20 μm). Many of the pigment-bearing cells were astrocytes (**d**, H&E, *bar* 15 μm). The brown pigment stained black in Masson Fontana preparations (**e**, *bar* 15 μm). NeuN staining of the resected temporal lobe showed subtle irregularities of cortical architecture with neuronal loss from layers II and III (**f**, *bar* 500 μm). This was accompanied by astrocytic gliosis (**g**, GFAP, *bar* 500 μm). At both sites, clusters of red granules were present (**h**, H&E, *bar* 20 μm). Electron microscopy demonstrated neuronal melanin in membrane-bound structures as well as individual melanosomes (**i**, *bar* 200 nm) and in association with vesicles of three electron densities, which included lipid (**j**, *bar* 300 nm). Melanophages contained large complex vesicles containing melanin granules (**k**, *bar* 2 μm). Melanin granules were also present within blood vessel walls (including endothelium) and within the lumen (**l**, *bar* 5 μm)

A 17-year-old male with multiple CMN had generalised tonic–clonic seizures from the age of 1 year and frequent, highly refractory partial seizures typical of temporal lobe epilepsy from the age of 8 years. Neurodevelopmental assessments revealed a normal IQ but below average verbal skills. Investigations including MRI (Fig. 1a), SPECT scanning and EEG localised the epileptogenic focus to the left amygdala, which in addition was smaller than the right. No other lesions were detected on MRI. In particular, the left temporal neocortex was unremarkable. He underwent an uncomplicated anterior temporal lobectomy at the age of 14 years, and has been seizure free for 36 months and off all antiepileptic medication for 24 months.

Surgical specimens from the left amygdala and left temporal lobe were examined. In neither was pigment apparent macroscopically. In the temporal lobe neocortex, there was a well-circumscribed area of abundant brown melanin pigment in layers I–IV of the cortex. The pigment was both within cells and free in the neuropil (Fig. 1b–d). The overlying leptomeninges were slightly thickened due to fibrosis but contained neither pigment nor melanocytes. The cortical pigment stained black on a Masson Fontana (MF) preparation (Fig. 1e), was bleached by potassium permanganate and did not fluoresce when exposed to ultraviolet light. Immunohistochemical staining for pre-melanosomes (HMB45) was positive in many of the pigment-bearing cells, including pyramidal neurones and cells that morphologically resembled astrocytes. Staining for tyrosinase was equivocal. The neurones were not dysmorphic and binucleate forms were not evident. Melanocytes were not apparent morphologically by light or electron microscopy and there was no immunostaining for the melanocyte marker microphthalmia transcription factor. Staining for calbindin, calretinin, NeuN and parvalbumin demonstrated subtle irregularities of cortical lamination with mild neuronal loss, most marked in layers II and III (Fig. 1f). There was astrocytic gliosis following the pattern of neuronal loss, demonstrated by staining for GFAP (Fig. 1g). In addition, there was subpial (Chaslin’s) gliosis. CD68 stained a minority of the pigment-bearing cells. The Ki67 proliferation index was very low and there was no cytological atypia, mitotic activity or other evidence of tumour. The appearances of the tissue from the left amygdala were very similar with large amounts of pigment both in the neuropil and in cells, including neurones. In this specimen the architecture was less organised, in keeping with the site. Many of the pigmented cells were in the parenchyma adjacent to vessels. Of note, in both specimens, there were occasional clusters of small brown–red granules that were often associated with aggregates of extracellular pigment but were not stained on the MF preparation (Fig. 1h).

To explore the nature of the melanin deposited in neurones, electron microscopy was undertaken. This showed melanin within neurones (Fig. 1i, j), melanophages (Fig. 1k) and blood vessels, including the endothelium and the lumina (Fig. 1l). A range of morphological patterns of melanin was noted. In some neurones, there were individual rounded membrane-bound melanosomes (Fig. 1i). However, in others, the neuronal melanin consisted of membrane-bound vesicles of differing electron density with distinct internal structure and often associated with small lipid droplets (Fig. 1j). The melanin containing organelles measured 0.4–1.2 µm in the neurones. Vesicles containing multiple granules of melanin were prevalent in the melanophages (Fig. 1k) but were not seen in neurones. Melanin granules in the melanophages measured <0.1–1.5 µm. There was no significant lipofuscin.

In summary, our case shows melanin deposition in neurones and glia in the absence of a significant melanocytic lesion. The neocortical lesion was associated with a subtle disturbance of cortical architecture in which there was mild loss of neurones from layers II and III. The remainder of the underlying laminar pattern was preserved and there was marked astrocytic gliosis, suggesting that these abnormalities may be a secondary abnormality. While the architectural changes are relatively subtle, they could be considered as a focal cortical dysplasia, Type IIId [2]. Only one of the histopathological lesions was visible on MRI, possibly due to the small area of the abnormality. This may explain the apparent discordant clinical and radiological findings in some individuals with NCM [14, 18].

Most previous histopathological reports of NCM have suggested that brain involvement is secondary to overlying and invasive leptomeningeal disease [16]. However, histological studies of parenchymal involvement of the amygdala without pigmentation of the overlying meninges have been reported [4, 5, 7, 11, 19, 20]. In these cases, the pigmentation was evident macroscopically and, where described, the pigment was usually in nests of melanocytes. In one case, in addition to melanocytic nests, neurones, including dysmorphic and binucleate forms, contained pigment [11].

In contrast, we describe two anatomically separate lesions in the mesial temporal lobe and neocortex in which parenchymal pigment was present within astrocytes and neurones in the absence of melanocytes. In our case, there was no evidence of a melanocytic component as judged by morphology, electron microscopy or immunohistochemistry. While we cannot exclude the presence of a small population of melanocytes, there is no evidence of nests of melanocytes similar to the previously described cases. While this is likely to indicate a lack of melanocytic proliferation, there is the intriguing possibility that this component was present and subsequently regressed, as may occur in the skin [13].

The ultrastructure of the melanin in neurones showed a wide range of morphological variation from electron-dense membrane-bound vesicles, similar to those seen in eumelanin production, to structures which show the three levels of electron density that has been described in neuromelanin but with rather more defined membranes than is sometimes described in neuromelanin [8]. The pattern of melanin production in these neurones is clearly very abnormal, differing from normal cutaneous melanin, normal neuromelanin and from melanosomes within CNS tumours [3, 6]. The mechanisms driving melanin production in the CNS component of NCM appear distinct and warrant further investigation.

Our case illustrates the difficulty of considering NCM as a neural crest disorder (‘neurocristopathy’) as it demonstrates abnormalities in both the neural crest and neural tube-derived cells. This is in keeping with the occasional observation of CNS malformations in NCM [15].
